# Do endurance athletes need repeat sweat sodium testing after heat acclimation? Separating physiological adaptation from measurement variability

**DOI:** 10.3389/fphys.2026.2002403

**Published:** 2026-10-20

**Authors:** He XiaoTian, YoonJung Wook, Zhang Sen, Jiang LiFang

**Affiliations:** 1 Woosuk University, Wanju, Republic of Korea; 2 Pingdingshan University, Henan, China

**Keywords:** endurance athletes, heat acclimation, measurement variability, personalized hydration, smallest detectable change, sudomotor function, sweat sodium concentration, sweat testing

## Abstract

Heat acclimation lowers the sodium concentration of sweat, a quantity widely measured to individualize fluid and electrolyte replacement in endurance athletes. Whether it should be repeated during or after acclimation remains unsettled. This Mini Review argues that the question is not whether the adaptation is real, since it reproduces across protocols and decades, but whether a second measurement can resolve it. Studies place the effect between roughly 9 and 23 mmol/L, or approximately 15 mmol/L at a matched local sweat rate, with a protocol-dependent time course: conservation detectable from the third day, before sweat rate rises, plateauing by the sixth day in field conditions but still falling at 10 days under laboratory acclimation. Against that signal, raising exercise intensity from 45% to 65% of maximal oxygen uptake shifts whole-body sweat sodium concentration by approximately 20 mmol/L, while collection site, technique, sample handling, and sodium intake add further variance. Analytical instrumentation is not the limiting step; procedural and biological variation is. Two interpretations follow: one treats serial testing as necessary to track a fast, heterogeneous adaptation, the other notes that the concentration predicts neither hydration status nor sweating rate, does not by itself stratify hyponatremia risk, and so rarely changes a decision. They differ in the quantity they estimate, not in their reading of the evidence. Repeat testing is defensible only under a reproduced heat stress protocol, reported as whole-body sodium loss rate, and interpreted against a smallest detectable change not yet established for acclimation monitoring; retest intervals remain hypotheses.

## 1 Introduction

Evaporation of sweat is the principal avenue of heat loss when endurance athletes train or compete in the heat, and the water and electrolytes lost with it bear on both performance and safety (Périard et al., 2021). Sweat sodium concentration is the quantity most often measured to characterize those losses, and in applied practice, it is used to individualize fluid and sodium replacement (Baker, 2017; Barnes et al., 2019). It is also a sudomotor marker of heat acclimation: repeated exercise-heat exposure raises sweat rate while lowering sweat sodium concentration, so that evaporative capacity improves as electrolyte loss per unit of sweat falls (Périard et al., 2015; Tyler et al., 2016).

That combination invites a practical question. If acclimation reliably lowers sweat sodium concentration, and if the measurement itself carries appreciable variability, should endurance athletes be tested repeatedly during or after an acclimation program? One interpretation holds that the adaptation is rapid and heterogeneous between athletes, so that serial measurement is the only way to keep a replacement plan current. Another interpretation deems that when procedural variance is uncontrolled, repeated testing risks reading noise as adaptation, and that sweat sodium concentration has no established relationship with the outcomes it is invoked to manage (Baker and Wolfe, 2020).

This Mini Review takes a position between those interpretations: the adaptation is not in doubt, but its resolvability by a second measurement is. We first establish the magnitude and time course of the acclimation effect, set it against the documented sources of variance in a sweat sodium number, examine what each interpretation is right about, and identify the gaps that must close before a retest interval can be specified from evidence rather than convention.

## 2 The adaptation: magnitude, time course, and modifiers

The direction of the effect has been consistent for four decades, and its locus is reasonably well defined. Kirby and Convertino (1986) acclimated 10 men over 10 consecutive days and found that both sweat sodium content and plasma aldosterone concentration fell, while the ratio of sweat sodium reabsorbed to plasma aldosterone rose; what increased was the ductal response to the hormone rather than the hormone itself. In eight men completing 10 days of exercise in the heat, Buono et al. (2007) showed that acclimation lowered the *y*-intercept of the relationship between sweat sodium concentration and sweat rate by 15 mmol/L without altering its slope, which places the change in ductal reabsorptive capacity rather than in flow. Chinevere et al. (2008) reported a fall from 54.5 ± 16.2 to 36.2 ± 7.2 mmol/L over 10 days while whole-body sweat rate rose by only approximately 6% and not significantly, which excludes dilution as the explanation.

Sodium conservation is also the earliest sudomotor adaptation to appear. Klous et al. (2020) found that sweat sodium and chloride concentrations fell to approximately 60% of day 1 values by day 10 at both arm and back, with conservation established from day 3, whereas local sweat rate did not increase until day 8 on the arm and day 7 on the back; potassium concentration was unchanged, indicating separate regulation of the two cations. At a 2-week training camp in natural heat, Karlsen et al. (2015) observed a fall from 75 ± 22 to 52 ± 24 mmol/L within the first 5 to 6 days with no further change thereafter, while power output continued to improve across the camp. That dissociation matters for the retest question: the marker can saturate while the phenotype it indexes is still developing.

Decay is equally informative. Klous et al. (2021) found that the sodium-conserving adaptation was no longer detectable after 28 days without heat exposure, and that 5 days of re-acclimation restored it, with controlled hyperthermia conserving more sodium than hot water immersion or no re-exposure. Cubel et al. (2024) lowered sweat sodium concentration by 14.1 mmol/L across 5 weeks of acclimation in elite cyclists and found performance and total hemoglobin mass back at baseline 2 weeks after withdrawal; the wider decay and re-induction literature was mapped early in trained athletes (Armstrong and Maresh, 1991) and has since been synthesized by meta-analysis (Daanen et al., 2018).

Magnitude depends on the stimulus and, less than is often assumed, on the athlete. Isothermic conditioning targeting a gastrointestinal temperature of at least 38.5°C reduced thigh-predicted sweat sodium concentration by 9 mmol/L (95% confidence interval 5 to 13 mmol/L), significantly more than a military acclimatization program in tropical natives (Tan et al., 2022). Age did not blunt the response: 8 days of acclimation lowered sweat sodium concentration similarly in young men and in highly fit and normally fit older men, the age difference appearing instead in sweat output per gland (Inoue et al., 1999). In tropic-dwelling men already exposed to passive heat, 14 days of exercise acclimation improved thermoregulatory and hematological markers without changing exercise sweat sodium concentration (Saat et al., 2005), a reminder that this marker does not always move with the rest of the phenotype. **Table 1** summarizes these studies.

**Table 1:** Sweat sodium concentration responses to heat acclimation: participants, protocol, magnitude, and time course.

Study	Participants	Acclimation protocol	Sweat [Na^+^] response	Time course and notes
Kirby and Convertino (1986)	10 men	10 days; 2 h/day at 45% of maximal oxygen uptake; 40 °C, 45% RH	Concentration and total content decreased from day 1 to day 10	Plasma aldosterone also fell, while the ratio of sweat sodium reabsorbed to plasma aldosterone rose; resting plasma volume increased 12%
Inoue et al. (1999)	Young men, highly fit older men, normally fit older men	8 days; 90 min/day cycling at approximately 35% of maximal oxygen uptake; 43°C, 30% RH	Decreased similarly in all three groups	Sweat output per gland lower in older men; local sweat rates unchanged by acclimation
Saat et al. (2005)	16 tropic-dwelling men	14 days; 60 min/day at 60% of maximal oxygen uptake; 31.1 °C, 70% RH	Unchanged during exercise; increased at rest	Thermoregulatory and hematological markers improved; passive heat exposure alone did not confer full acclimatization
Buono et al. (2007)	8 men	10 days of exercise in the heat	*y*-intercept of the concentration versus sweat rate relation reduced by 15 mmol/L; slope unchanged	Locates the adaptation in ductal reabsorption rather than in sweat flow
Chinevere et al. (2008)	8 men	10 days; up to 100 min/day treadmill walking; 45 °C, 20% RH	54.5 ± 16.2 to 36.2 ± 7.2 mmol/L (approximately 34% lower)	Whole-body sweat rate rose only approximately 6% and not significantly; arm glove, first 30 min of exercise
Karlsen et al. (2015)	Trained cyclists	2-week training camp in natural heat	75 ± 22 to 52 ± 24 mmol/L	Fall within the first 5 to 6 days, with no further change by day 13 (tests on days 1, 6, and 13); sweat rate increased 20% ± 15%; power output continued to improve
Buono et al. (2018)	4 adults	7 days; 2 h/day treadmill walking; 40 °C, 40% RH	Downward shift of the concentration versus sweat rate relation on days 3, 5, and 7	Linear decline at a fixed sweat rate (*r* = −0.50)
Klous et al. (2020)	8 participants	10 days controlled hyperthermia; 33 °C, 65% RH	Fell to approximately 60% of day 1 values at arm and back	Conservation from day 3, before local sweat rate rose (day 8 arm, day 7 back); potassium unchanged
Klous et al. (2021)	15 participants	10 days controlled hyperthermia, 28 days decay, 5 days re-acclimation	Sodium, chloride, and lactate 25% to 34% lower (arm) and 21% to 27% lower (back) after acclimation; no longer detectable after decay (sampled only at 28 days); re-established after re-acclimation	Active re-acclimation conserved more sodium than hot water immersion or control
Tan et al. (2022)	Tropical-native men (*n* = 17 per program)	2 weeks; 10 sessions of isothermic conditioning or military acclimatization, or 6 sessions of interval training	Thigh-predicted concentration reduced by 9 mmol/L (95% confidence interval 5 to 13 mmol/L) after isothermic conditioning	Isothermic conditioning (target gastrointestinal temperature at least 38.5 °C) exceeded the military program; no significant reduction after the military or interval programs
Cubel et al. (2024)	20 male elite cyclists	5 weeks of heat acclimation	Reduced by 14.1 mmol/L from pre to post	Performance and total hemoglobin mass returned to baseline 2 weeks after withdrawal
Willmott et al. (2021)	1 adult with cystic fibrosis	10 sessions; 90 min at 40 °C, 40% RH	70 to 52 mmol/L (reduced by 18 mmol/L)	Adaptations retained at least 7 days; no cramping on follow-up testing; single case

Values are mean ± standard deviation where reported in the source. RH, relative humidity.

## 3 What a sweat sodium number actually measures

A reported sweat sodium concentration is a composite of secretion rate, ductal reabsorption, anatomical site, collection technique, sample handling, analytical method, and recent sodium intake. Each has been quantified, and several rival the acclimation effect in size (**Table 2**).

**Table 2:** The heat acclimation signal set against documented sources of variance in a sweat sodium measurement.

Source of variation	Reported magnitude	Implication for a repeat test	Evidence
Heat acclimation (the signal to be detected)	9 to 23 mmol/L between studies; 15 mmol/L at a matched local sweat rate; substantial adaptation within the first week in some protocols, still declining at day 10 in others	The quantity a retest is intended to resolve	(Buono et al., 2007; Karlsen et al., 2015; Klous et al., 2020; Tan et al., 2022)
Exercise intensity (45% versus 65% of maximal oxygen uptake)	Whole-body 32.6 ± 14.3 versus 52.7 ± 14.6 mmol/L; total sodium loss 659 ± 340 versus 1,565 ± 590 mg	Exceeds the acclimation effect; workload must be fixed across tests	(Baker et al., 2019)
Local sweat rate (flow dependence of ductal reabsorption)	Expected 26.5 to 49.7 mmol/L across a fivefold range of whole-body sweat rate	Sweat rate must be co-reported for a concentration to be interpretable	(Buono et al., 2007; Taylor and Machado-Moreira, 2013)
Anatomical site	Eccrine gland density 16 to 530 glands per square centimeter; regional values differ systematically from whole body; uncorrected chest and upper-back patches exceed whole-body values by a mean of 22 to 23 mmol/L	Identical site and patch placement required; convert regional values with validated equations before calculating sodium loss rate	(Baker, 2017; Baker et al., 2019, 2020; Taylor and Machado-Moreira, 2013)
Collection and sample handling technique	ICC 0.93 to 0.96 between extraction and analysis routes; mean differences small but usually significant	Technique must not change between tests	(Baker et al., 2014)
Analytical instrument	Within-instrument ICC at least 0.999 and CV no more than 2.6%; between instruments SEE 2.8 to 3.8 mmol/L and CV 3.9 to 12.3%	Not the limiting source of error; consequences judged trivial in most circumstances	(Goulet et al., 2017)
Normal variability in sweat sodium concentration	Approximately ±12%	Context for expected fluctuation only; not a smallest detectable change or decision threshold	(Goulet et al., 2017)
Between-trial test–retest reliability	CV 10.4% (closed pouch) and 23% to 24% (portable analyzers); TE 8 mmol/L (patch conductivity) and 5.6 mmol/L (predicted whole-body values, 5-h cycling)	SDC = 1.96 × √2 × TE, approximately 15 mmol/L for the 5.6 mmol/L estimate; no SDC established for acclimation heat stress tests	(Goulet et al., 2026; Hayden et al., 2004; Relf et al., 2019; Zhao et al., 2026)
Habitual sodium intake	Controlled intakes of 348.4, 174.1, and 66.3 mmol/day; largest reduction in sweat sodium secretion on the lowest intake, with potentiated aldosterone	Diet must be documented or standardized before a retest	(Allsopp et al., 1998)
Wearable monitoring	Local sweat rate increased 35% by monitor versus 26% to 48% by patch; conductivity fell 21% to 25%	Conductivity measures total ionic content rather than sodium; sodium-selective sensors compared with analyzers only within single sessions	(Dyshko et al., 2024; Relf et al., 2020)

ICC, intraclass correlation coefficient; CV, coefficient of variation; SEE, standard error of the estimate; SDC, smallest detectable change; TE, typical error.

Flow dependence is intrinsic. Because ductal reabsorption is rate-limited, concentration rises with local sweat rate (Buono et al., 2007), and across a fivefold range of whole-body sweat rate, the expected sodium concentration spans 26.5 to 49.7 mmol/L (Taylor and Machado-Moreira, 2013). Exercise intensity therefore moves the number directly: raising work rate from 45% to 65% of maximal oxygen uptake raised whole-body sweat sodium concentration from 32.6 ± 14.3 to 52.7 ± 14.6 mmol/L, and total sodium loss from 659 ± 340 to 1,565 ± 590 mg, in the same participants (Baker et al., 2019). A repeat test performed at a different workload can therefore manufacture or erase an apparent adaptation larger than the adaptation itself.

Site and technique add further structure. Eccrine gland density varies across the body surface from 16 to 530 glands per square centimeter, with corresponding differences in regional sweat flow (Taylor and Machado-Moreira, 2013), and regional concentrations differ systematically from whole-body values, which is why normative datasets normalize patch measurements to whole-body equivalents (Baker, 2017; Barnes et al., 2019). Uncorrected, chest and upper-back patches overestimated whole-body concentration by a mean of 22 to 23 mmol/L, whereas validated prediction equations reduced mean bias to 5 mmol/L or less (Baker et al., 2020); sodium loss rate should therefore combine whole-body sweat rate with a measured or validly converted whole-body concentration. Handling and extraction contribute smaller but detectable bias: extraction methods and analytical routes agreed closely, yet mean differences from the reference route were usually significant (Baker et al., 2014; **Table 2**).

The analytical step is not the weak link. Comparing five instruments on 70 samples, Goulet et al. (2017) found within-instrument coefficients of variation of 2.6% or less and between-instrument standard errors of 2.8 to 3.8 mmol/L (**Table 2**); set against a normal variability in sweat sodium concentration of approximately ±12%, they judged the resulting imprecision trivial in most exercise circumstances. The variance that threatens a repeat test is procedural and biological, not instrumental.

Habitual sodium intake is a confounder specific to serial testing. With daily intake controlled at 348.4, 174.1, and 66.3 mmol, the reduction in sweat sodium secretion during heat exposure was greatest on the low-intake condition, with potentiated aldosterone secretion (Allsopp et al., 1998). An athlete who changes sodium intake between two tests changes the result independently of acclimation state.

Wearable monitors address the logistical burden but not the inferential one. A wearable sweat rate monitor tracked a 35% rise in local sweat rate across a 5-day program against 26% to 48% by absorbent patch, and sweat conductivity fell by 21% to 25% (Relf et al., 2020). Conductivity, however, reflects total ionic content rather than sodium specifically.

The consequence is inferential rather than technical. Interpreting a change between two tests requires the smallest detectable change (SDC) of the measurement under the protocol used, derived from the typical error (TE) of repeated tests; at 95% confidence, SDC = 1.96 × √2 × TE. The ±12% cited above describes expected fluctuation, not an SDC. Test–retest estimates exist for some exercise-heat protocols (Hayden et al., 2004; Relf et al., 2019; Zhao et al., 2026; **Table 2**); over two 5-h cycling trials in the heat, predicted whole-body concentration had a between-trial TE of 5.6 mmol/L (Goulet et al., 2026), which would imply an SDC of approximately 15 mmol/L for that protocol, comparable to the acclimation effect itself. We identified no SDC for the heat stress tests used to monitor acclimation.

## 4 Two interpretations, and what each is right about

The case for repeat testing rests on three observations. First, the trajectory is concentrated in the induction period, although its timing varies: conservation was detectable from day 3 and continued to fall on day 10 in one controlled protocol (Klous et al., 2020), but appeared to plateau by days 5 to 6 at a field training camp (Karlsen et al., 2015). If the aim is to describe that trajectory, or to estimate cumulative sodium loss across a program, measurement must be concentrated in that window. The corollary is that, where the response plateaus early, a test after the program may add little over one late in the first week, and that a marker that saturates while the broader phenotype continues to develop cannot serve as a running index of acclimation state. Serial testing is therefore informative about sudomotor sodium handling specifically, and only while that handling is still changing. Second, the magnitude is heterogeneous. Group mean reductions range from 9 mmol/L in a field program (Tan et al., 2022) through 14.1 mmol/L over 5 weeks of laboratory acclimation (Cubel et al., 2024) to 23 mmol/L at a training camp (Karlsen et al., 2015), and the between-participant standard deviations at that camp, 22 mmol/L before and 24 mmol/L after, were as large as the mean change itself. A group mean is therefore a poor substitute for an individual value when the purpose is individualized replacement (Rivera-Brown and Quiñones-González, 2020). Third, the measurement can be consequential in identifiable subgroups: an adult with cystic fibrosis and a baseline concentration of 70 mmol/L who had previously cramped during exercise in the heat reduced it by 18 mmol/L over 10 sessions, retained the adaptations for at least 7 days, and showed no cramping on follow-up heat stress testing (Willmott et al., 2021).

The case against rests on the weakness of the link between the number and any outcome. Sweat and blood concentrations have not been shown to correspond for most solutes, and sweat electrolyte concentrations predict neither hydration status nor sweating rate (Baker and Wolfe, 2020). The third international consensus conference on exercise-associated hyponatremia attributes the disorder principally to fluid intake in excess of losses combined with non-osmotic vasopressin-mediated water retention; thus, a sweat sodium value does not, by itself, stratify risk (Hew-Butler et al., 2015). Because acclimation increases sweat loss, acclimated athletes need to pay particular attention to fluid replacement (Sawka and Montain, 2000), which means preventing large deficits rather than replacing all losses regardless of thirst; the consensus statement recommends drinking to thirst (Hew-Butler et al., 2015).

Framed as a disagreement about evidence, the dispute is intractable; framed as a disagreement about estimands, it largely resolves. The first interpretation estimates a sudomotor phenotype, for which sweat sodium concentration is a valid and sensitive index. The second asks whether the estimate changes a decision, for which the relevant quantity is the rate of sodium loss, the product of whole-body sweat rate and whole-body concentration, which differs several-fold between sports and carries the practical consequence (Barnes et al., 2019). Both positions are compatible with the same data, and both are served by the same change in practice: hold the protocol fixed, report loss rate rather than concentration alone, and pre-specify the change worth acting on. We identified no study that has manipulated acclimation-induced changes in sweat sodium concentration and measured performance or heat illness as an outcome; hence, the proposition that tracking the marker improves either remains an untested inference.

## 5 Research gaps and future directions

Five gaps follow from the preceding sections, each with a matching priority. First, reliability. TEs exist for a few exercise-heat protocols (Section 3), but we identified no SDC under the heat stress tests used to monitor acclimation, the quantity needed to interpret a retest; reliability studies reporting both, at a stated confidence level, are the highest priority. Second, decay kinetics. Decay and re-induction have been characterized chiefly for core temperature, heart rate, and sweat rate (Daanen et al., 2018), whereas for sodium conservation, the direct evidence amounts to a single 28-day observation in 15 participants, with no intermediate time points (Klous et al., 2021); dose–response decay studies would allow a retest interval to be specified rather than assumed. Third, populations. Women, adolescents, older athletes, and clinical groups remain under-represented: menstrual cycle phase was recorded but not controlled in that decay study (Klous et al., 2021), the female-specific evidence comes mainly from short-term programs in which sweat rate rose and sweat sodium chloride fell without the time course being resolved (Mee et al., 2018), the cystic fibrosis evidence is one case and one small early study with discordant results (Orenstein et al., 1984; Willmott et al., 2021), and seasonal acclimatization in healthy adults has been reviewed without being linked to sweat sodium trajectories (Brown et al., 2022). Adequately powered studies documenting cycle phase are needed. Fourth, outcomes. Small trials have compared plans matched to measured sweat and sodium losses with *ad libitum* drinking, with mixed results (Ayotte and Corcoran, 2018; Emerson et al., 2017), but we identified none randomizing athletes to sweat-test-guided versus standardized sodium replacement with performance or illness endpoints; such a trial would settle the utility question that currently divides the field. Fifth, mechanism. Ductal transporter-level evidence in human acclimation remains indirect, inferred from concentration and flow relationships and from hormone ratios (Buono et al., 2007; Kirby and Convertino, 1986); direct human ductal work would ground the interpretation of the concentration as a transport phenotype.

Two developments would change the calculus. Continuous wearable sensing of sodium specifically would replace serial discrete tests with a trajectory and remove much of the procedural variance. We identified no such device validated for this purpose: a sodium-selective sensor has been compared with a commercial analyzer only within single sessions (Dyshko et al., 2024), and the only wearable we found evaluated across acclimation measured sweat rate, not sodium (Relf et al., 2020). More efficient induction protocols, whether self-regulated intermittent training that achieved comparable adaptation with 23% less training volume (Roussey et al., 2021) or programs built around a target core temperature (Tan et al., 2022), shorten the window within which repeated measurement could plausibly be informative.

## 6 Discussion

Whether endurance athletes need repeat sweat sodium testing after heat acclimation cannot be answered by yes or no, because the answer is a property of the measurement protocol rather than of the athlete. The adaptation is genuine, early and modest in size, of the order of 15 mmol/L at a matched sweat rate. The measurement can move by a comparable or larger amount for reasons unrelated to acclimation, principally exercise intensity, collection site, technique, and recent sodium intake, while the analytical instruments themselves are precise. A second test that does not reproduce the first is therefore uninterpretable, and one that does reproduce it may report a change that had largely run its course within the first week in the field, although concentrations continued to fall through 10 days of controlled laboratory acclimation (Karlsen et al., 2015; Klous et al., 2020).

On the present evidence, the defensible position is conservative, and the timings below are provisional, research-informed examples rather than evidence-based intervals. Characterize the athlete once while unacclimatized, under a heat stress test that can be reproduced, and report the whole-body rate of sodium loss alongside sweat rate rather than concentration alone. When the explicit aim is to describe the induction trajectory or estimate cumulative sodium loss, one additional measurement, for example, around day 7, could be considered, although the decline plateaued by days 5 to 6 in the field yet continued to day 10 in the laboratory. Retest when the premises change: after a substantial alteration in training intensity or environment, or after a period without heat exposure long enough for decay to be plausible. The only direct observation of decay in sodium conservation, a small study sampling only at 28 days (Klous et al., 2021), cannot show when the adaptation was lost; a 4-week interval is therefore a hypothesis to be tested by the dose–response studies called for above (Daanen et al., 2018), not an evidence-based trigger. No numerical threshold for acting on a change can yet be recommended, because the ±12% variability cited by Goulet et al. (2017) is not an SDC and none has been established for acclimation monitoring (Section 3). Clinical and high-risk subgroups are the exception to this conservatism, because where an elevated baseline concentration is itself the problem, as in cystic fibrosis, serial measurement shows whether the intended effect occurs rather than tracking an incidental marker (Orenstein et al., 1984; Willmott et al., 2021). Otherwise, treat sweat sodium concentration as an index of sudomotor function rather than as a clinical endpoint (Baker and Wolfe, 2020).

What would overturn this position is specific and achievable: a reliability estimate showing that a standardized protocol resolves changes appreciably smaller than the 15 mmol/L matched-rate effect, together with a trial in which replacement guided by serial testing improved an outcome that matters to athletes. We identified neither. Until both exist, the burden of proof lies with repeated testing rather than against it, and the effort that repeat testing would consume is better spent standardizing the first test.

## References

[B1] Allsopp A. J. Sutherland R. Wood P. Wootton S. A. (1998). The effect of sodium balance on sweat sodium secretion and plasma aldosterone concentration. Eur. J. Appl. Physiol. 78, 516–521. doi: 10.1007/s004210050454 9840406

[B2] Armstrong L. E. Maresh C. M. (1991). The induction and decay of heat acclimatisation in trained athletes. Sports Med. 12, 302–312. doi: 10.2165/00007256-199112050-00003 1763248

[B3] Ayotte D. Jr. Corcoran M. P. (2018). Individualized hydration plans improve performance outcomes for collegiate athletes engaging in in-season training. J. Int. Soc Sports Nutr. 15, 27. doi: 10.1186/s12970-018-0230-2 29866199 PMC5987390

[B4] Baker L. B. (2017). Sweating rate and sweat sodium concentration in athletes: A review of methodology and intra/interindividual variability. Sports Med. 47, 111–128. doi: 10.1007/s40279-017-0691-5 28332116 PMC5371639

[B5] Baker L. B. De Chavez P. J. D. Ungaro C. T. Sopeña B. C. Nuccio R. P. Reimel A. J. . (2019). Exercise intensity effects on total sweat electrolyte losses and regional vs. whole-body sweat [Na+], [Cl-], and [K+. Eur. J. Appl. Physiol. 119, 361–375. doi: 10.1007/s00421-018-4048-z 30523403 PMC6373370

[B6] Baker L. B. Nuccio R. P. Reimel A. J. Brown S. D. Ungaro C. T. De Chavez P. J. D. . (2020). Cross-validation of equations to predict whole-body sweat sodium concentration from regional measures during exercise. Physiol. Rep. 8, e14524. doi: 10.14814/phy2.14524 32748563 PMC7399373

[B7] Baker L. B. Ungaro C. T. Barnes K. A. Nuccio R. P. Reimel A. J. Stofan J. R. (2014). Validity and reliability of a field technique for sweat Na+and K+analysis during exercise in a hot-humid environment. Physiol. Rep. 2, e12007. doi: 10.14814/phy2.12007 24793982 PMC4098735

[B8] Baker L. B. Wolfe A. S. (2020). Physiological mechanisms determining eccrine sweat composition. Eur. J. Appl. Physiol. 120, 719–752. doi: 10.1007/s00421-020-04323-7 32124007 PMC7125257

[B9] Barnes K. A. Anderson M. L. Stofan J. R. Dalrymple K. J. Reimel A. J. Roberts T. J. . (2019). Normative data for sweating rate, sweat sodium concentration, and sweat sodium loss in athletes: An update and analysis by sport. J. Sports Sci. 37, 2356–2366. doi: 10.1080/02640414.2019.1633159 31230518

[B10] Brown H. A. Topham T. H. Clark B. Smallcombe J. W. Flouris A. D. Ioannou L. G. . (2022). Seasonal heat acclimatisation in healthy adults: A systematic review. Sports Med. 52, 2111–2128. doi: 10.1007/s40279-022-01677-0 35460514 PMC9388416

[B11] Buono M. J. Ball K. D. Kolkhorst F. W. (2007). Sodium ion concentration vs. sweat rate relationship in humans. J. Appl. Physiol. 103, 990–994. doi: 10.1152/japplphysiol.00015.2007 17600161

[B12] Buono M. J. Kolding M. Leslie E. Moreno D. Norwood S. Ordille A. . (2018). Heat acclimation causes a linear decrease in sweat sodium ion concentration. J. Therm. Biol. 71, 237–240. doi: 10.1016/j.jtherbio.2017.12.001 29301696

[B13] Chinevere T. D. Kenefick R. W. Cheuvront S. N. Lukaski H. C. Sawka M. N. (2008). Effect of heat acclimation on sweat minerals. Med. Sci. Sports Exerc. 40, 886–891. doi: 10.1249/mss.0b013e3181641c04 18408609

[B14] Cubel C. Fischer M. Stampe D. Klaris M. B. Bruun T. R. Lundby C. . (2024). Time-course for onset and decay of physiological adaptations in endurance trained athletes undertaking prolonged heat acclimation training. Temperature. 11, 350–362. doi: 10.1080/23328940.2024.2383505 39583901 PMC11583594

[B15] Daanen H. A. M. Racinais S. Périard J. D. (2018). Heat acclimation decay and re-induction: A systematic review and meta-analysis. Sports Med. 48, 409–430. doi: 10.1007/s40279-017-0808-x 29129022 PMC5775394

[B16] Dyshko K. Nicodemus M. P. Otterstetter R. Ghadimi H. Daniels S. Fulmer M. S. . (2024). Evaluation of a wearable fabric-based sensor for accurate sodium determination in sweat during exercise. Eur. J. Appl. Physiol. 124, 1347–1353. doi: 10.1007/s00421-023-05364-4 38019318

[B17] Emerson D. M. Torres-McGehee T. M. Emerson C. C. LaSalle T. L. (2017). Individual fluid plans versus ad libitum on hydration status in minor professional ice hockey players. J. Int. Soc Sports Nutr. 14, 25. doi: 10.1186/s12970-017-0183-x 28775674 PMC5539891

[B18] Goulet E. D. Asselin A. Gosselin J. Baker L. B. (2017). Measurement of sodium concentration in sweat samples: Comparison of 5 analytical techniques. Appl. Physiol. Nutr. Metab. 42, 861–868. doi: 10.1139/apnm-2017-0059 28407476

[B19] Goulet E. D. B. Jeker D. Claveau P. Deshayes T. A. Pancrate T. Abed M. E. F. . (2026). Temporal stability, reproducibility and predictability of whole-body sweat sodium concentration during prolonged cycling in the heat with ad libitum and programmed drinking. Nutrients 18, 989. doi: 10.3390/nu18060989 41901164 PMC13029115

[B20] Hayden G. Milne H. C. Patterson M. J. Nimmo M. A. (2004). The reproducibility of closed-pouch sweat collection and thermoregulatory responses to exercise-heat stress. Eur. J. Appl. Physiol. 91, 748–751. doi: 10.1007/s00421-004-1057-x 14985998

[B21] Hew-Butler T. Rosner M. H. Fowkes-Godek S. Dugas J. P. Hoffman M. D. Lewis D. P. . (2015). Statement of the third international exercise-associated hyponatremia consensus development conference, carlsbad, california 2015. Clin. J. Sport Med. 25, 303–320. doi: 10.1097/jsm.0000000000000221 26102445

[B22] Inoue Y. Havenith G. Kenney W. L. Loomis J. L. Buskirk E. R. (1999). Exercise- and methylcholine-induced sweating responses in older and younger men: Effect of heat acclimation and aerobic fitness. Int. J. Biometeorol. 42, 210–216. doi: 10.1007/s004840050107 10232057

[B23] Karlsen A. Nybo L. Nørgaard S. J. Jensen M. V. Bonne T. Racinais S. (2015). Time course of natural heat acclimatization in well‐trained cyclists during a 2‐week training camp in the heat. Scandinavian Med. Sci. Sports. 25, 240–249. doi: 10.1111/sms.12449 25943675

[B24] Kirby C. R. Convertino V. A. (1986). Plasma aldosterone and sweat sodium concentrations after exercise and heat acclimation. J. Appl. Physiol. 61, 967–970. doi: 10.1152/jappl.1986.61.3.967 3759782

[B25] Klous L. De Ruiter C. Alkemade P. Daanen H. Gerrett N. (2020). Sweat rate and sweat composition during heat acclimation. J. Therm. Biol. 93, 102697. doi: 10.1016/j.jtherbio.2020.102697 33077118

[B26] Klous L. de Ruiter C. Alkemade P. Daanen H. Gerrett N. (2021). Sweat rate and sweat composition following active or passive heat re-acclimation: A pilot study. Temperature. 8, 90–104. doi: 10.1080/23328940.2020.1826287 33553508 PMC7849678

[B27] Mee J. A. Peters S. Doust J. H. Maxwell N. S. (2018). Sauna exposure immediately prior to short-term heat acclimation accelerates phenotypic adaptation in females. J. Sci. Med. Sport. 21, 190–195. doi: 10.1016/j.jsams.2017.06.024 28739443

[B28] Orenstein D. M. Henke K. G. Green C. G. (1984). Heat acclimation in cystic fibrosis. J. Appl. Physiol. Respir. Environ. Exerc. Physiol. 57, 408–412. doi: 10.1152/jappl.1984.57.2.408 6381439

[B29] Périard J. D. Eijsvogels T. M. H. Daanen H. A. M. (2021). Exercise under heat stress: Thermoregulation, hydration, performance implications, and mitigation strategies. Physiol. Rev. 101, 1873–1979. doi: 10.1152/physrev.00038.2020 33829868

[B30] Périard J. D. Racinais S. Sawka M. N. (2015). Adaptations and mechanisms of human heat acclimation: Applications for competitive athletes and sports. Scandinavian Med. Sci. Sports. 25, 20–38. doi: 10.1111/sms.12408 25943654

[B31] Relf R. Eichhorn G. Waldock K. Flint M. S. Beale L. Maxwell N. (2020). Validity of a wearable sweat rate monitor and routine sweat analysis techniques using heat acclimation. J. Therm. Biol. 90, 102577. doi: 10.1016/j.jtherbio.2020.102577 32479383

[B32] Relf R. Willmott A. Flint M. S. Beale L. Maxwell N. (2019). Reliability of a wearable sweat rate monitor and routine sweat analysis techniques under heat stress in females. J. Therm. Biol. 79, 209–217. doi: 10.1016/j.jtherbio.2018.12.019 30612681

[B33] Rivera-Brown A. M. Quiñones-González J. R. (2020). Normative data for sweat rate and whole-body sodium concentration in athletes indigenous to tropical climate. Int. J. Sport Nutr. Exerc. Metab. 30, 264–271. doi: 10.1123/ijsnem.2019-0299 32454460

[B34] Roussey G. Bernard T. Fontanari P. Louis J. (2021). Heat acclimation training with intermittent and self-regulated intensity may be used as an alternative to traditional steady state and power-regulated intensity in endurance cyclists. J. Therm. Biol. 98, 102935. doi: 10.1016/j.jtherbio.2021.102935 34016357

[B35] Saat M. Sirisinghe R. G. Singh R. Tochihara Y. (2005). Effects of short-term exercise in the heat on thermoregulation, blood parameters, sweat secretion and sweat composition of tropic-dwelling subjects. J. Physiol. Anthropol. Appl. Hum. Sci. 24, 541–549. doi: 10.2114/jpa.24.541 16237263

[B36] Sawka M. N. Montain S. J. (2000). Fluid and electrolyte supplementation for exercise heat stress. Am. J. Clin. Nutr. 72, 564S–572S. doi: 10.1093/ajcn/72.2.564s 10919961

[B37] Tan S. C. C. Ang W. H. Lim L. S. X. Low I. C. C. Lee J. K. W. (2022). Efficacy of isothermic conditioning over military-based heat acclimatization and interval training in tropical native males. Med. Sci. Sports Exerc. 54, 1925–1935. doi: 10.1249/mss.0000000000002991 35787594 PMC9632943

[B38] Taylor N. A. Machado-Moreira C. A. (2013). Regional variations in transepidermal water loss, eccrine sweat gland density, sweat secretion rates and electrolyte composition in resting and exercising humans. Extrem. Physiol. Med. 2, 4. doi: 10.1186/2046-7648-2-4 23849497 PMC3710196

[B39] Tyler C. J. Reeve T. Hodges G. J. Cheung S. S. (2016). The effects of heat adaptation on physiology, perception and exercise performance in the heat: A meta-analysis. Sports Med. 46, 1699–1724. doi: 10.1007/s40279-016-0538-5 27106556

[B40] Willmott A. G. Holliss R. Saynor Z. Corbett J. Causer A. J. Maxwell N. S. (2021). Heat acclimation improves sweat gland function and lowers sweat sodium concentration in an adult with cystic fibrosis. J. Cyst. Fibros. 20, 485–488. doi: 10.1016/j.jcf.2020.07.013 32758394

[B41] Zhao X. McKenna Z. J. Wierick S. C. Perez R. I. Sellner R. Allen B. M. . (2026). Reliability and validity of portable sweat sodium analyzers during exercise in the heat. Appl. Physiol. Nutr. Metab. 51, 1–9. doi: 10.1139/apnm-2025-0482 42190283

